# Medication adherence rates in patients with ocular inflammatory disease

**DOI:** 10.3389/fmed.2026.1745392

**Published:** 2026-02-17

**Authors:** Zhi Rui John Chew, Lin-Yi Clare Cheng, Rupesh Agrawal, Su Ling Ho, Xin Wei, Zheng Xian Thng

**Affiliations:** 1 Yong Loo Lin School of Medicine, National University of Singapore, Singapore, Singapore; 2 Department of Ophthalmology, Tan Tock Seng Hospital, National Healthcare Group Eye Institute, Singapore, Singapore

**Keywords:** adherence, adherence rates, barriers to adherence, medication adherence, ocular inflammatory disease, targeted educational intervention, understanding, uveitis

## Abstract

**Aim:**

To evaluate medication adherence patterns in patients with ocular inflammatory disease.

**Methods:**

This cross-sectional study involved patients with ocular inflammatory disease who completed a questionnaire assessing demographics, disease characteristics, understanding of condition and medication regimen, self-reported adherence and methods utilized to improve adherence. Accuracy of self-reported responses were validated against electronic hospital records. Univariate and multivariate analyses were performed to identify factors associated with adherence and understanding.

**Results:**

Of the 75 patients with ocular inflammatory disease, 14 (18.7%) were evaluated to be highly adherent to their prescribed medications, despite 33 (44%) reporting no perceived barriers to adherence. 38 (50.6%) were unable to recall the purpose of their medications, despite 59 (78.6%) reporting that the purpose of their medications was clearly explained to them. The ability to accurately recall their ocular inflammatory diagnosis (*p* = 0.041) was independently associated with good adherence rates. 19 (25.3%) displayed good understanding of their ophthalmic condition and medication regimen. A higher income was independently associated with good understanding of their condition and medication regimen (*p* = 0.032).

**Conclusion:**

Medication adherence in ocular inflammatory disease is suboptimal. Our results indicate a discordance between explained and recalled medication and condition information, impacting adherence negatively. Targeted educational interventions may be required to enhance patients’ understanding and retention of key information regarding their ocular inflammatory disease and medication regimen to improve adherence.

## Introduction

Ocular Inflammatory Disease (OID) comprises a variety of sight-threatening conditions such as uveitis and scleritis, and is a leading cause of preventable vision loss (1, 2), associated with 10–15% of blindness worldwide. OID can be broadly categorized into infectious and non-infectious etiologies. While infectious etiologies result from an identifiable microbial cause, non-infectious etiologies are often immune-mediated and can either occur in isolation or in relation to a systemic inflammatory or autoimmune condition (1). To prevent progression of disease, local or systemic medications are prescribed in order to induce and maintain long-term remission. Treatment regimens may differ from patient to patient depending on the severity and control of disease, often requiring several medications administered at varying frequencies throughout the day, hence posing a significant medication burden on patients who suffer from OID, with up to 50% of patients on more than four daily medications (1, 3).

Adherence to medication can be defined as the extent to which a person’s behavior in taking medication corresponds with agreed recommendations from a healthcare provider (4). Non-adherence may limit the benefit of the prescribed treatment, resulting in poor control of disease, manifesting as more frequent flares, irreversible vision loss and functional impairment (5, 6).

Previous studies have shown that polypharmacy, understanding of disease and medication side effects are amongst some significant barriers that affect adherence to medications in patients with ophthalmic conditions such as glaucoma and ocular inflammatory disease (3, 7, 8). While there has been substantial literature published on medication adherence in glaucoma (9–12), few studies explore adherence in ocular inflammatory disease (6–8).

This study aims to understand and determine the rates, contributory factors, associations and key barriers of drug adherence in patients with ocular inflammatory disease, contextualized in the local Asian population. The findings will help inform and guide the development of targeted interventions to address specific challenges faced by these patients.

## Materials and methods

### Ethics

This cross-sectional study was approved by the local ethics committee of National Healthcare Group (NHG) Domain Specific Review Board (DSRB) (Approval number: 2024-4250) and adhered to the tenets of the Declaration of Helsinki. Written consent was obtained from all participants that were recruited between April to May 2025.

### Participants

This was a single-center cross-sectional study at a tertiary eye center in Singapore. Patients were interviewed consecutively at an ocular inflammatory clinic in our center. Patients that were currently undergoing a prescribed treatment regimen with local and/or systemic therapy were recruited. Eligibility was assessed by a trained researcher or a board-certified ophthalmologist prior to enrolling the patient to the study and obtaining informed consent. Exclusion criteria were age below 21, first visits, severe cognitive impairment and inability to provide informed consent as well as spoken languages other than English or Mandarin.

### Data collection

A total of 77 patients consented to participate in the study, 75 were confirmed to be eligible after excluding patients who were no longer on active treatment for their ocular inflammatory condition. A survey questionnaire comprising of 44 questions was administered face-to-face in a private room prior to their appointment with their attending physician by a trained researcher not involved in their care. The survey questionnaire design included components such as the patient’s demographics, understanding of their condition, understanding of medication regimen, perceived adherence and methods attempted to improve adherence. Accuracy of patient recollection of their condition, medication regimen and control of disease was subsequently evaluated by the same interviewer via referring to their electronic health records.

The primary outcome of the study was the self-reported medication adherence rates in patients with ocular inflammatory disease. Secondary outcomes include understanding and recollection of their condition and medication regimen, evaluating the impact of understanding on adherence, associations with demographics factors on both understanding and adherence and the effectiveness of any prior interventions used to improve adherence.

### Study tool

The questionnaire was developed and drafted by one of the authors based on existing literature (13–17) and frameworks. The questionnaire comprises of 5 parts: patient’s demographics, understanding of their condition, understanding of medication regimen, perceived adherence and methods attempted to improve adherence.

Adherence was measured using the 8-item Morisky Medication Adherence Scale (MMAS-8) (18–20). It is a validated self-reporting tool comprising a set of 8 questions to assess medication-taking behavior. Utilization of the validated scoring algorithm gave a total score ranging from 0 to 8. While the original MMAS-8 categorizes patients into good (= 8), moderate (6–7) and low (< 6) adherence, we dichotomized patients into only two categories, good adherence (= 8) and poor adherence (< 8). This approach was adopted to reduce discriminatory performance as prior evidence suggests that the traditional cutoff of ≥ 6 demonstrates limited sensitivity and moderate specificity in identifying adherent patients (21).

Understanding was measured using a set of nine questions, with five allocated for understanding of condition and four allocated for understanding of medication regimen. Patients were required to recall details pertaining to their condition as well as their medication regimen, and were scored based on their accuracy of recollection. Their responses were retrospectively tallied with their electronic health record to assess the accuracy of recollection, scoring “1” point for every accurate recollection and “0” points for every inaccurate recollection. The cutoffs values were aligned conceptually to the 8-item Morisky Medication Adherence Scale (MMAS-8) scoring system. Patients who scored “<8” or “8–9” were considered to have poor and good understanding of their condition and medication regimen respectively.

The interview was concluded by an open-ended question for the patients to elaborate on any challenges in understanding or adherence or provide suggestions for possible interventions to improve patient adherence.

### Sample size determination

This study was designed to be an exploratory, descriptive cross-sectional study. No formal power size calculation was performed, as there were limited published data on effect sizes and adherence rates in patients with ocular inflammatory disease to inform reliable power estimates. The sample size was therefore determined based on feasibility and the number of eligible patients attending the clinic at the during the study period.

### Statistical methods

Adherence was measured using MMAS-8 and categorized into good and poor adherence according to established cutoffs. Understanding was measured using our novel questionnaire and categorized into good and poor understanding. Data was analyzed using IBM SPSS Statistics, Version 29.0.2.0 (IBM Corp., Armonk, NY, United States).

Categorical variables, such as demographic and clinical characteristics, were summarized as frequencies and percentages, while continuous variables, such as age, were reported as mean ± standard deviation (SD). The association between adherence and demographic factors, as well as patients’ understanding of their condition and treatment, was assessed using Chi-square test. For variables with more than two categories and small cell counts, associations were assessed using Fisher’s exact test, and only global *p*-values were reported. Odds ratios and 95% confidence intervals were presented only for binary variables with an appropriate reference category. Variables with *p*- < 0.20 in univariate analysis were included in a multivariable logistic regression model. Adjusted odds ratios (aOR) with 95% confidence intervals (95% CI) were calculated. A *p-*value of < 0.05 was considered statistically significant.

## Results

A total of 75 patients were included in the study, with 41 (54.6%) female and 34 (45.3%) male patients and a mean (± SD) age of 58 (14.50). Patient demographics and the clinical characteristics of ocular inflammatory disease are shown in Tables 1, 2 respectively. 59 (78.7%) patients were on topical treatment, with all using topical eyedrops, including patients on topical treatment alone and those on combination therapies. The remaining patients were on systemic treatment, with 10 (13.3%) on oral treatment alone and 3 (4.0%) on subcutaneous treatment alone. A total of 57 (76%) of patients were on multiple medications for their ocular inflammatory condition, some requiring a up to eight medications as shown in Table 3.

**TABLE 1:** Patient demographics.

Variable	Category	Count (N%)
Age	Mean (Std)	58.32 (± 14.5)
Gender	Female	41 (54.6%)
	Male	34 (45.3%)
Language	English	42 (56.0%)
	Mandarin	33 (44.0%)
Ethnicity	Malay	6 (8.0%)
	Chinese	63 (84.0%)
	Indian	5 (6.6%)
	Others	1 (1.3%)
Marital status	Single	16 (21.6%)
	Married	57 (77.0%)
	Widowed/divorced	1 (1.3%)
Education	None	4 (5.3%)
	Primary school	13 (17.3%)
	Secondary school	25 (33.3%)
	Pre university	16 (21.3%)
	Bachelors	15 (20.0%)
	Masters	2 (2.7%)
Income/month	No income (retired or unemployed)	32 (42.7%)
	<$2,000	4 (5.3%)
	$2,000–$2,999	1 (1.3%)
	$3,000–$3,999	9 (12.0%)
	≥$4,000	17 (22.6%)
	Do not wish to disclose	12 (16.0%)
Household members	Alone	10 (13.3%)
	Spouse	24 (32.0%)
	Spouse and children	28 (37.3%)
	Siblings only	4 (5.3%)
	Parents only	5 (6.6%)
	Children only	3 (4.0%)
	Others	1 (1.3%)
Medical insurance	Private	49 (65.3%)
	Government	22 (29.3%)
	None or unsure	4 (5.3%)

**TABLE 2:** Ocular inflammatory conditions of participants.

Anatomic location/etiology	Count (N%)
Anterior uveitis: Undifferentiated anterior uveitis (16) RA-related uveitis (11) HLA-B27 related uveitis (4) Fuchs Heterochromic iridocyclitis (2) Posner–Schlossman syndrome (1) Psoriatic uveitis (2) Phacoantigenic uveitis (1) Sjogren-related uveitis (1)	38 (50.7%)
Intermediate uveitis: Undifferentiated intermediate uveitis (6)	6 (8.0%)
Posterior uveitis: Undifferentiated retinal vasculitis (6) Undifferentiated choroiditis (3) Undifferentiated posterior uveitis (3) CMV retinitis (2) AZOOR (1) Tuberculosis-related uveitis (1)	16 (21.3%)
Panuveitis: Behcet’s disease (3) Undifferentiated panuveitis (4)	7 (9.3%)
Scleritis/sclerouveitis (8)	8 (10.7%)
Total	75 (100.0%)
**Duration of disease**	**Count (N%)**
Chronic (> 6 months)	64 (85.3%)
Acute (< 6 months)	11 (14.7%)
Total	75 (100.0%)

**TABLE 3:** Medication and appointment frequency.

Variable	Category	Count (N%)
Number of medications	1.00	18 (24.0%)
	2.00	24 (32.0%)
	3.00	17 (22.6%)
	4.00	7 (9.3%)
	5.00	5 (6.6%)
	6.00	2 (2.6%)
	8.00	2 (2.6%)
Mode/s of administration	Topical only	37 (49.3%)
	Oral Only	10 (13.3%)
	Subcutaneous only	3 (4.0%)
	Intravenous only	1 (1.3%)
	Topical + Oral	20 (26.7%)
	Topical + Subcutaneous	2 (2.7%)
	Oral + Subcutaneous	1 (1.3%)
	Oral + Intravenous	1 (1.3%)
Type of medication	Steroids only	29 (38.7%)
	Steroids + others*	36 (48.0%)
	Steroid-sparing agent only	1 (1.3%)
	Others only	9 (12.0%)
Follow-up appointment frequency	≤ 2 months	22 (29.7%)
	2–6 months	31 (41.9%)
	≥ 6 months	18 (24.3%)
	Yearly	3 (4.1%)

*Includes non-steroidal immunosuppressive agents, intra-ocular pressure lowering agents, anti-viral, anti-biotic or anti-fungal medication.

**FIGURE 1:**
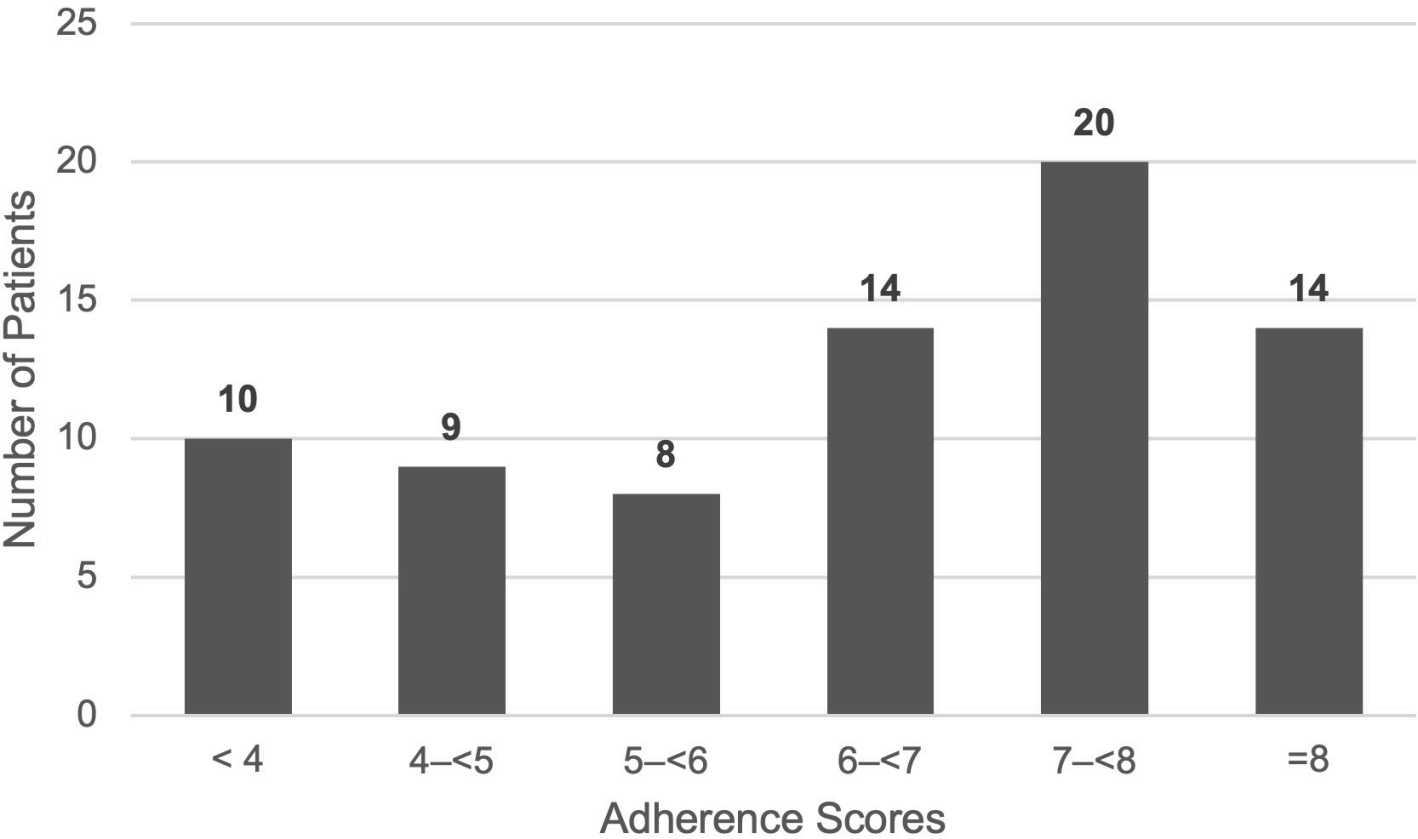
Histogram of adherence scores.

With regard to self-reported adherence to the prescribed medication regimen (Table 4), only 14 (18.7%) patients were evaluated to have good adherence, despite 33 (44.0%) patients reporting no perceived barriers to adherence, 71 (94.7%) patients reporting that instructions on how to take medications were clear and 54 (72.0%) patients reporting that they found their medication regimen to be uncomplicated as shown in Table 5. 65 (86.6%) patients only miss one dose or less per week on average. The most common reasons provided for non-adherence was forgetfulness (38.6%) and inconvenience (10.6%). A histogram of adherence scores (Figure 1) shows that, although only 14 patients met the cutoff for “Good Adherence,” an additional 34 patients scored within the 6– < 8 point range, suggesting a substantial portion of patients with borderline adherence.

**TABLE 4:** Responses for self-reported adherence.

(©2007 Donald E. Morisky) Self-Reported Adherence (Morisky Medication Adherence Scale (MMAS-8)*		Count (N%)
Do you sometimes forget to take your medications?	No	35 (46.6%)
	Yes	40 (53.3%)
People sometimes miss taking their medications for reasons other than forgetting. Thinking over the past two weeks, were there any days when you did not take your medicine?	No	59 (78.6%)
	Yes	16 (21.3%)
Have you ever cut back or stopped taking your medication without telling your doctor, because you felt worse when you took it?	No	62 (82.6%)
	Yes	13 (17.3%)
When you travel or leave home, do you sometimes forget to bring along your medicine?	No	68 (90.6%)
	Yes	7 (9.3%)
Did you take your medications yesterday?	No	14 (18.6%)
	Yes	61 (81.3%)
When you feel your condition is under control, do you sometimes stop taking your medicine?	No	63 (84.0%)
	Yes	12 (16.0%)
Taking medication every day is a real inconvenience for some people. Do you ever feel hassled about sticking to your treatment plan?	No	47 (62.6%)
	Yes	28 (37.3%)
How often do you have difficulty remembering to take all your medicine?	All the time	0 (0.0%)
	Usually	0 (0.0%)
	Sometimes	7 (9.3%)
	Once in a while	23 (30.6%)
	Never	45 (60.0%)
**Adherence grouping**		**Count (N%)**
Poor Adherence (< 8)		61 (81.3%)
Good Adherence (= 8)		14 (18.7%)
Total		75 (100.0%)
How often do you miss doses of your medication in a typical week?	Never	46 (61.3%)
	Once	19 (25.3%)
	Twice	6 (8.0%)
	Thrice	3 (4.0%)
	Four times	1 (1.3%)
**Barriers to adherence**		**Count (N%)**
NIL		33 (44.0%)
Forget		29 (38.6%)
Inconvenience		8 (10.6%)
Cost		2 (2.6%)
Side Effects		3 (3.9%)

*The MMAS-8 Scale, content, name and trademarks are protected by US copyright and trademark laws. Permission for use of the scale and its coding is required. A license agreement is available from MMAR, LLC., www.moriskyscale.com.

**TABLE 5:** Responses for understanding of medications and condition.

Variable	Response	Count (N%)
Recalls number of medications*	No	17 (22.7%)
	Yes	58 (77.3%)
Recalls name of medications*	No	50 (66.6%)
	Yes	25 (33.3%)
Recalls dosage of medications*	No	19 (25.3%)
	Yes	56 (74.6%)
Recalls purpose of medications*	No	38 (50.6%)
	Yes	37 (49.3%)
Was the purpose of your medications explained to you clearly?	No	16 (21.3%)
	Yes	59 (78.6%)
Do you find your medication regimen to be complicated?	No	54 (72.0%)
	Yes	21 (28.0%)
Do you find that the instructions on how to take your medications are clear?	No	4 (5.3%)
	Yes	71 (94.7%)
Are you aware of the complications of your condition?*	No	16 (21.3%)
	Yes	59 (78.6%)
Recalls ocular inflammatory condition*	No	57 (76.0%)
	Yes	18 (24.0%)
Recalls previous ocular surgery or laser*	No	8 (10.7%)
	Yes	67 (89.3%)
Recalls comorbidities*	No	17 (22.7%)
	Yes	58 (77.3%)
Recalls duration of condition since diagnosis*	No	25 (33.3%)
	Yes	50 (66.6%)
Belief of cure	Maintain	49 (65.3%)
	Fully Cure	16 (21.6%)
	Not sure	7 (9.4%)
	Will worsen	2 (2.7%)
**Understanding grouping**		**Count (N%)**
Poor understanding (< 8)		56 (74.7%)
Good understanding (≥ 8)		19 (25.3%)
Total		75 (100.0%)

*Responses used in scoring of understanding.

**FIGURE 2:**
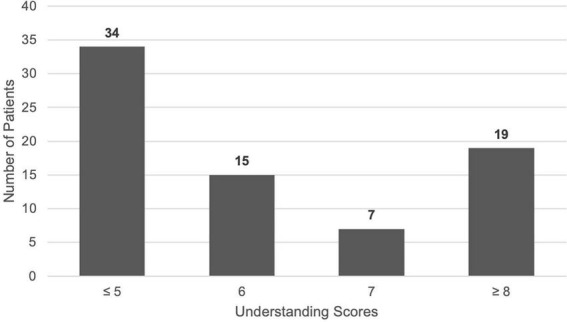
Histogram of understanding scores.

With regard to understanding of condition and medication regimen (Table 5), only 19 (25.3%) patients were evaluated to have good understanding. Notably, 38 (50.6%) patients being unable to accurately recall the purpose of their medications, despite 59 (78.6%) patients reporting that the rationale for their prescriptions had been clearly explained. However, 58 (77.3%) patients were able to accurately recall the number of prescribed medications and 59 (78.6%) were aware of the potential complications of poor disease control. A histogram of understanding scores (Figure 2) shows that only 7 patients scored close to the “Good Understanding” cutoff, with the majority of 34 patients scoring ≤ 5 points, suggesting generally limited understanding of condition and medications.

We found that 48 (64.0%) patients did not use any form of reminder system to improve adherence. Among those who did, the most common tool used was mobile phone alarm reminders, reported by 20 (26.7%) patients. Most patients did not involve family members, caregivers, or friends in assisting with reminders; 70 (93.3%) relied solely on themselves to remember to take their medications, while only 5 (6.6%) enlisted the help of others. Interestingly, 60 (80%) received only verbal education about their condition and medication regimen from their doctor or pharmacist, with just 12 (16%) receiving additional resources such as pamphlets or written instructions. In addition, 3 (4.0%) patients reported receiving no educational resources from their healthcare providers.

Multivariate analysis (Table 6) showed that accurate recall of their ocular inflammatory diagnosis was significantly associated with good adherence (*p* = 0.041). On univariate analysis, accurate recall of the purpose of medications (*p* = 0.001) and accurate recall of duration of condition since diagnosis (*p* = 0.027) were each significantly associated with good adherence (Table 6). However, these associations did not remain significant after adjustment for confounders. No statistically significant associations with adherence were found for demographic factors, polypharmacy, mode of application, use of reminder systems, or prior educational resources provided (Table 7 and Supplementary Tables 1, 2).

**TABLE 6:** Logistic regression results for the effect of understanding on adherence.

Variable	Category	Good adherence (*n* = 14)	Poor adherence (*n* = 61)	Univariate model		Multivariate model	
				OR (95% CI)	*p-*value	OR (95% CI)	*p-*value
Recalls ocular inflammatory condition	No	1 (7.1%)	24 (39.3%)	3.451 (0.899–13.241)	**0.027**	9.054 (1.092, 75.092)	**0.041**
	Yes (Ref)	13 (92.9%)	37 (60.7%)	1 (Ref)	–	–	–
Recalls duration of condition since diagnosis	No	1 (7.1%)	24 (39.3%)	8.432 (1.035–68.711)	**0.027**	6.125 (0.701–52.521)	0.101
	Yes (Ref)	13 (92.9%)	37 (60.7%)	1 (Ref)	–	–	–
Aware of complications of disease	No	3 (21.4%)	13 (21.3%)	NA	1		
	Yes (Ref)	11 (78.6%)	48 (78.7%)	1 (Ref)	–		
Recalls comorbidities	No	2 (14.3%)	15 (24.6%)	3.110 (0.641–15.098)	0.502		
	Yes (Ref)	12 (85.7%)	46 (75.4%)	1 (Ref)	–		
Recalls previous ocular surgery or laser	No	2 (14.3%)	6 (10.0%)	0.655 (0.117–3.647)	0.638		
	Yes (Ref)	12 (85.7%)	55 (90.0%)	1 (Ref)	–		
Recalls number of medications	No	4 (28.6%)	13 (21.3%)	7.200 (0.886–58.531)	0.724		
	Yes (Ref)	10 (71.4%)	48 (78.7%)	1 (Ref)	–		
Recalls name of medications	No	8 (57.1%)	42 (68.9%)	1.658 (0.505–5.445)	0.531		
	Yes (Ref)	6 (42.9%)	19 (31.1%)	1 (Ref)	–		
Recalls dosage of medications	No	3 (21.4%)	16 (26.2%)	NA	1		
	Yes (Ref)	11 (78.6%)	45 (73.8%)	1 (Ref)	–		
Recalls purpose of medications	No	6 (42.9%)	32 (52.5%)	15.300 (3.203–73.087)	**0.001**	1.546 (0.433–5.649)	0.495
	Yes (Ref)	8 (57.1%)	29 (49.3%)	–	–		
Finds instructions clear	No	0 (0.0%)	4 (6.6%)	NA	1		
	Yes (Ref)	14 (100.0%)	57 (93.4%)	1 (Ref)	–		
Understands the purpose of medication	No	5 (35.7%)	11 (18.0%)	6.585 (0.807–53.709)	0.161		
	Yes (Ref)	9 (64.3%)	50 (82.0%)	1 (Ref)	–		
Finds medication regimen complicated	No	12 (85.7%)	42 (68.9%)	0.893 (0.276–2.889)	0.324		
	Yes (Ref)	2 (14.3%)	19 (31.1%)	1 (Ref)	–		
Belief of cure	Cannot cure	4 (28.6%)	19 (31.7%)	1.023 (0.364–2.872)	0.966		
	Cure/maintain (Ref)	10 (71.4%)	41 (68.3%)	1 (Ref)	–		

Bold values indicate statistically significant.

**TABLE 7:** Univariate logistic regression results for the effect of methods on adherence.

Variable	Category	Good adherence (*n* = 14)	Poor adherence (*n* = 61)	OR (95% CI)	*p*-value
Usage of reminder systems	None	7 (50.0%)	41 (67.2%)	2.050 (0.632–6.646)	0.419
	Any reminder systems- alarm, calander etc. (Ref)	7 (50.0%)	20 (32.8%)	1 (Ref)	–
Reminders by people	Yes	0 (0.0%)	5 (8.2%)	NA	0.298
	No (Ref)	14 (100.0%)	56 (91.8%)	1 (Ref)	–
Educational resources by healthcare providers	None	1 (7.1%)	2 (3.3%)	0.400 (0.023–6.848)	0.527
	Verbal	11 (78.6%)	49 (80.3%)	0.891 (0.171–4.653)	0.891
	Verbal + one visual (Ref)	2 (14.3%)	10 (16.4%)	1 (Ref)	–

Multivariate analysis (Table 8) also found that higher income was significantly associated with good understanding of their condition and medication regimen (*p* = 0.032). On univariate analysis, English speaking patients (*p* = 0.008), singlehood (*p* = 0.028), education level above university (*p* = 0.005) and younger age (< 70 years; *p* = 0.001) were each significantly associated with good understanding (Table 8). However, these associations did not remain significant after adjustment for confounders.

**TABLE 8:** Logistic regression results for the effect of patient demographics on understanding.

Variable	Category	Poor understanding (*n* = 56)	Good Understanding (*n* = 19)	Univariate model		Multivariate model	
				OR (95% CI)	*p-*value	OR (95% CI)	*p-*value
Age	>70	22 (39.3%)	0 (0.0%)	NA	**0.001**	NA	0.944
	<70 (Ref)	34 (60.7%)	19 (100.0%)	1 (Ref)	–	1 (Ref)	–
Gender	Female	32 (57.1%)	9 (47.4%)	1.481 (0.521–4.211)	0.460		
	Male (Ref)	24 (42.9%)	10 (52.6%)	1 (Ref)	–		
Language	Mandarin	30 (53.6%)	3 (15.8%)	6.154 (1.611–23.509)	**0.008**	2.577 (0.423–15.697)	0.305
	English (Ref)	26 (46.4%)	16 (84.2%)	1 (Ref)	–	1 (Ref)	–
Ethnicity	Malay	4 (7.1%)	2 (10.5%)	0.681 (0.114–4.077)	0.674		
	Indian	4 (7.1%)	1 (5.3%)	1.362 (0.142–13.096)	0.789		
	Others	1 (1.8%)	0 (0.0%)	NA	NA		
	Chinese (Ref)	47 (83.9%)	16 (84.2%)	1 (Ref)	–		
Marital status	Single	9 (16.1%)	8 (42.1%)	0.263 (0.083–0.837)	**0.028**	0.521 (0.116–2.347)	0.396
	Married (Ref)	47 (83.9%)	11 (57.9%)	1 (Ref)	–	1 (Ref)	–
Education	Before university	48 (85.6%)	10 (52.6%)	5.400 (1.674–17.416)	**0.005**	2.033 (0.510–8.103)	0.314
	University and above (Ref)	8 (14.3%)	9 (47.4%)	1 (Ref)	–	1 (Ref)	–
Income*	< $3,000/month	34 (70.8%)	3 (20.0%)	9.714 (2.372–39.788)	**0.001**	5.72 (1.159, 28.220)	**0.032**
	> $3,000/month (Ref)	14 (29.2%)	12 (80.0%)	1 (Ref)	–	1 (Ref)	–
Household members	Lives Alone	8 (14.3%)	2 (10.5%)	1.417 (0.273–7.342)	0.678		
	Lives with Others (Ref)	48 (85.7%)	17 (89.5%)	1 (Ref)	–		
Insurance	Private or Corporate	36 (64.3%)	13 (68.4%)	0.923 (0.088–9.682)	0.947		
	Government	17 (30.4%)	5 (26.3%)	1.133 (0.096–13.440)	0.921		
	None/Unsure (Ref)	3 (5.4%)	1 (5.3%)	1 (Ref)	–		

*Patients who did not disclose income or highest education level were excluded from the analysis. Bold values indicate statistically significant.

A total of eight feedback responses were received from the interviewed patients. Five patients reported difficulty in reading the small font on the topical eyedrop bottle. One patient reported difficulty in understanding the prescribed steroid tapering schedule, another reported being unaware of a medication side effect and one expressed not receiving adequate explanation regarding their ocular inflammatory condition.

## Discussion

Our study shows that medication adherence among patients with ocular inflammatory disease in the local population is suboptimal, with fewer than one in five patients demonstrating good adherence according to our survey. A key observation from our study is the discordance between the poor medication adherence and patients’ lack of reported barriers to adherence. A possible explanation could be that many patients are unaware of their own obstacles and barriers to being adherent to their medication regimen. Earlier conclusions in ocular inflammatory disease and other chronic eye diseases such as glaucoma suggest that poor self-efficacy, complex medication regimens and poor understanding are indeed major barriers to optimal control (3, 9–12). However, although only a minority of patients achieved good adherence, nearly one-third scored just below the cutoff, suggesting that mild or occasional non-adherence may be common and may potentially improve with targeted interventions. Interestingly, our survey identified three patients who reported receiving no educational resources, either verbal or written. Although this represents a small proportion of the cohort, this finding is concerning as patient education is fundamental to medication adherence and effective disease control (6). Two potential explanations may account for this observation. Firstly, these patients may truly have never received any form of education from their healthcare provider regarding their condition and medication regimen, although this is less likely given the multiple layers of patient education routinely provided at our institution by ophthalmologists, nurses and pharmacists. Alternatively and more plausibly, these patients had previously received education but do not recall it. Both scenarios are concerning as the former would represent a lapse in care provision, while the latter suggests inadequate reinforcement of previously provided education and retention of educational information. Regardless of the underlying cause, this finding further supports regular, reinforced educational interventions to improve patient understanding and improve adherence amongst patients with ocular inflammatory disease.

Another key observation is the discordance between the perceived clarity of information provided and the accuracy of patient recall. Although more than three-quarter of our patient population reported that the purpose of their medications was clearly explained, slightly less than half were able to recall it when prompted. In addition, only one-quarter of patients demonstrated good overall understanding of both their disease and medication regimen. Prior studies by Sandberg et al and Watson et al on recall and retention show that providing information does not equal to information retention without reinforcement and tailored counseling (22, 23), hence highlighting an opportunity for structured patient education and reinforcement.

Contrary to expectations, polypharmacy and mode of application did not significantly affect adherence in our cohort. However, previous studies by Tsai et al. and Gatwood et al. have also shown that medication regimen complexity does not always translate into poorer adherence (11, 12), even though it might be expected to complicate daily routines. Instead, we found that the strongest factor associated with good adherence was accurate recall of their ocular inflammatory condition. This suggests that disease insight may be more important than the practical burden of a complex medication regimen. The histogram (Figure 2) findings further support this, with nearly half of the patients scoring less than five out of a total of nine points for understanding, reflecting a broader lack of insight and understanding regarding their condition and medications. This reinforces the need to emphasize holistic patient education, as well as to encourage regular recollection with the goal of improving understanding and retention of information received (24).

Steroid-related side effects are a commonly reported and well-recognized barrier to medication adherence in ocular inflammatory disease (25, 26). In our cohort, almost nine in ten patients were currently being treated with steroids, with thirteen patients reporting that they had previously discontinued their medications because they “felt worse” after taking them as captured by the MMAS-8 questionnaire. This finding reflects the real-world challenges of long-term corticosteroid use, where the need for effective disease control and medication tolerability must be balanced carefully. It highlights the importance for patient education and counseling regarding potential side effects, regular monitoring and timely introduction of steroid-sparing immunosuppressants to mitigate non-adherence and achieve long-term disease control.

Interestingly, our study also found that income level was independently associated with better understanding of condition and medication regimen. This is consistent with existing understanding that socioeconomic advantage is linked to improved health literacy and greater access to healthcare resources (27). This highlights a disparity that may partly explain why some patients are better able to recall their condition and medication regimen compared to others. Although other demographic variables such as language, marital status, education level and age were significantly associated with understanding on univariate analysis, these relationships were not sustained after multivariate adjustment. This is likely due to the inter-correlation between these factors which are closely related to health literacy. Interestingly, we also found that there were no demographic variables associated with adherence, perhaps suggesting that adherence challenges are not confined to specific patient subgroups, but instead are potentially multi-factorial and modifiable. Additionally, the modest sample size and reduced power may be insufficient to detect independent effects.

Our results on methods used to improve adherence were limited. Notably, only half of the patients in the good adherence group reported using reminders such as tele-reminders or alarms, with its effectiveness being unconclusive. This could be partly due to the fact that self-setting alarms and reminders requires an element of intrinsic motivation and do not overcome deeper barriers such as poor understanding or low motivation. A clinical trial done by Lai et al. found that automated dosing reminders have proven to be effective in improving adherence in patients with glaucoma (28). While the role of automated dosing reminders in improving adherence in patients with ocular inflammatory disease should be explored more extensively in the future, the findings from our study suggest that for these patients recruited in our study, intrinsic motivation remains a key driver of adherence compared to external tools. Qualitatively among patients that have good adherence, a key motivating factor is the fear of vision loss. This translates to a greater awareness and ownership toward the treatment of their condition, with the understanding that adherence to their prescribed medications is the necessary driver to ensure their vision does not deteriorate.

A common qualitative feedback from patients was the difficulty in reading the instructions on their prescribed topical eyedrops. Eyedrops come in small bottles, with the accompanying text often printed in very small font, posing challenges for patients with reduced visual acuity or age-related presbyopia. As a result, many patients must rely solely on their memory of verbal instructions given by their doctor or pharmacist, which can be especially difficult for elderly patients who may have impaired cognition or short-term memory. Existing literature on glaucoma suggests that providing clear written instructions or educational pamphlets (29, 30) can help bridge this gap by giving patients a physical visual reference, improving their understanding of their condition and medication regimen compared to relying only on recall.

In Singapore, sociocultural factors including language barriers, cultural beliefs about medication and health literacy play a crucial role in shaping medication adherence and disease understanding. Brief and non-tailored education and communication with healthcare professionals is an added obstacle in improving this. While not formally assessed in our study, these factors may influence adherence and understanding and should be a topic explored in future studies in order to develop culturally sensitive, person-centered interventions to improve patient adherence and understanding in Singapore’s multi-ethnic society.

In summary, our findings demonstrate that medication adherence among patients with ocular inflammatory disease remains suboptimal despite regular follow-up and counseling. Our study suggests that this gap is driven less by the complexity of treatment regimens and more by patients’ limited retention of essential information about their disease and medications. Notably, accurate recall of diagnosis rather than polypharmacy or mode of administration, was the strongest factor associated with good adherence, highlighting the central role of patient insight and understanding.

## Recommendation

Reinforced, structured patient education using simple but clear, repeated explanations in verbal and written format is key to addressing poor adherence and understanding in patients with ocular inflammatory disease. Ideally, these materials should be tailored to patients’ literacy level and language when possible. Large-print labels or supplementary printed pamphlets have the potential to address the common issue of small-print instructions on eyedrop bottles, particularly in patients with poor visual acuity.

Despite our study not finding any statistically significant impact from reminder systems, these should not be dismissed, and instead should be explored extensively in further studies. Practical aids such as visual dosing charts, automated reminder systems and family support are potential aides that may be effective in improving adherence to medications in patients with ocular inflammatory disease.

Bridging the gap between the information the patient receives and what they are able to retain is key to ensuring sustained, long-term understanding and adherence of their ocular inflammatory condition and medication regimen. When possible, healthcare providers should periodically assess the patients’ retention of crucial information pertaining to their condition and medications.

## Limitations

This study has several limitations that may affect the interpretation of the results. First, being a cross-sectional study relying on reported data, it is subject to recall bias, whereby participants inaccurately estimate or recall their medication regimen, condition and adherence, as well as Second, social desirability bias may have led some patients to overreport adherence in order to avoid appearing non-adherent to their prescribed medication. In addition, our study included English and Mandarin speaking patients, hence possibly resulting in underrepresentation of certain demographic of patients which may affect the results reported due to selection bias. Participants who chose to participate may perhaps be systematically different from patients who declined to participate, potentially leading to an overestimation of adherence rates due to selection bias as well. Treatment and disease related information was extracted from electronic health records, which may vary in consistency across different visits and providers. While objective information such as medication prescriptions and diagnosis can be reliably verified, subjective information such as prior education received relied on self-reporting and thus susceptible to recall bias.

Given the chronic nature of most ocular inflammatory conditions, the course of the disease often has quiescent and active periods, depending on the control and management of the disease. As such, this study does not account for the temporal fluctuations in adherence that may be the result of changing disease activity and medication burden on the patient. Our study’s relatively small sample size (*n* = 75) limits the statistical power for some subgroup analyses to detect significant associations, especially for variables with small cell counts. This may have contributed to wide confidence intervals and associated factors which do not reach statistically significant values, potentially underestimating the effect of each variable. Further studies with larger sample sizes will be needed to confirm these findings and improve generalizability.

## Data Availability

The raw data supporting the conclusions of this article will be made available by the authors, without undue reservation.

## References

[1] Maghsoudlou P Epps SJ Guly CM Dick AD. Uveitis in adults: a review. *JAMA*. (2025) 334:419–34. 10.1001/jama.2025.4358 40434762

[2] Miserocchi E Fogliato G Modorati G Bandello F. Review on the worldwide epidemiology of uveitis. *Eur J Ophthalmol*. (2013) 23:705–17. 10.5301/ejo.5000278 23661536

[3] Sun K Marshall R Frankland M Taylor A Montana C Crowell E Barriers to adherence with immunosuppressive therapy in patients with uveitis. *Ocul Immunol Inflamm*. (2025) 33:619–26. 10.1080/09273948.2024.2430709 39591521

[4] Osterberg L Blaschke T. Adherence to medication. *N Engl J Med*. (2005) 353:487–97. 10.1056/NEJMra050100 16079372

[5] Dick AD Tundia N Sorg R Zhao C Chao J Joshi A Risk of ocular complications in patients with noninfectious intermediate uveitis, posterior uveitis, or panuveitis. *Ophthalmology*. (2016) 123:655–62. 10.1016/j.ophtha.2015.10.028 26712559

[6] Dolz-Marco R Gallego-Pinazo R Díaz-Llopis M Cunningham ET Arévalo JF. Noninfectious uveitis: strategies to optimize treatment compliance and adherence. *Clin Ophthalmol*. (2015) 9:1477–81. 10.2147/OPTH.S36650 26316689 PMC4547652

[7] Sun K Marshall R Frankland M Taylor A Montana C Crowell E Barriers to adherence with clinic visits in patients with uveitis. *Ocul Immunol Inflamm*. (2025) 33:1243–7. 10.1080/09273948.2025.2456641 39834139

[8] Javidi H Poonit N Patel RP Barry RJ Rauz S Murray PI. Adherence to topical medication in patients with inflammatory eye disease. *Ocul Immunol Inflamm*. (2021) 29:890–5. 10.1080/09273948.2019.1699122 31944132

[9] Bott D Subramanian A Edgar D Lawrenson JG Campbell P. Barriers and enablers to medication adherence in glaucoma: a systematic review of modifiable factors using the Theoretical Domains Framework. *Ophthalmic Physiol Opt*. (2024) 44:96–114. 10.1111/opo.13245 37985237

[10] Newman-Casey PA Robin AL Blachley T Farris K Heisler M Resnicow K The most common barriers to glaucoma medication adherence: a cross-sectional survey. *Ophthalmology*. (2015) 122:1308–16. 10.1016/j.ophtha.2015.03.026 25912144 PMC4485580

[11] Gatwood J Brooks C Meacham R Abou-Rahma J Cernasev A Brown E Facilitators and barriers to glaucoma medication adherence. *J Glaucoma*. (2022) 31:31–6. 10.1097/IJG.0000000000001965 34772874

[12] Tsai JC. A comprehensive perspective on patient adherence to topical glaucoma therapy. *Ophthalmology.* (2009) 116(11 Suppl):S30–6. 10.1016/j.ophtha.2009.06.024 19837258

[13] Cabral AC Lavrador M Castel-Branco M Figueiredo IV Fernandez-Llimos F. Development and validation of a medication adherence Universal Questionnaire: the MAUQ. *Int J Clin Pharm.* (2023) 45:999–1006. 10.1007/s11096-023-01612-x 37329432 PMC10366321

[14] Larsen RE Pripp AH Krogstad T Johannessen Landmark C Holm LB. Development and validation of a new non-disease-specific survey tool to assess self-reported adherence to medication. *Front Pharmacol*. (2022) 13:981368. 10.3389/fphar.2022.981368 36569319 PMC9768604

[15] Lavsa SM Holzworth A Ansani NT. Selection of a validated scale for measuring medication adherence. *J Am Pharm Assoc*. (2011) 51:90–4. 10.1331/JAPhA.2011.09154 21247831

[16] Atsuta R To Y Sakamoto S Mukai I Kobayashi A Kinoshita A Assessing usability of the “Adherence Starts with Knowledge 20” (ASK-20) questionnaire for Japanese adults with bronchial asthma receiving inhaled corticosteroids long term. *Allergol Int*. (2017) 66:411–7. 10.1016/j.alit.2016.09.001 27712949

[17] Svarstad BL Chewning BA Sleath BL Claesson C. The Brief Medication Questionnaire: a tool for screening patient adherence and barriers to adherence. *Patient Educ Couns*. (1999) 37:113–24. 10.1016/s0738-3991(98)00107-4 14528539

[18] Berlowitz DR Foy CG Kazis LE Bolin LP Conroy MB Fitzpatrick P Effect of intensive blood-pressure treatment on patient-reported outcomes. *N Engl J Med*. (2017) 377:733–44. 10.1056/NEJMoa1611179 28834483 PMC5706112

[19] Bress AP Bellows BK King JB Hess R Beddhu S Zhang Z Cost-effectiveness of intensive versus standard blood-pressure control. *N Engl J Med*. (2017) 377:745–55. 10.1056/NEJMsa1616035 28834469 PMC5708850

[20] Krousel-Wood M Islam T Webber LS Re RN Morisky DE Muntner P. New medication adherence scale versus pharmacy fill rates in seniors with hypertension. *Am J Manag Care.* (2009) 15:59–66. 19146365 PMC2728593

[21] Moon SJ Lee WY Hwang JS Hong YP Morisky DE. Accuracy of a screening tool for medication adherence: a systematic review and meta-analysis of the Morisky Medication Adherence Scale-8. *PLoS One*. (2017) 12:e0187139. 10.1371/journal.pone.0187139 29095870 PMC5667769

[22] Sandberg EH Sharma R Sandberg WS. Deficits in retention for verbally presented medical information. *Anesthesiology*. (2012) 117:772–9. 10.1097/ALN.0b013e31826a4b02 22902965

[23] Watson PW McKinstry B. A systematic review of interventions to improve recall of medical advice in healthcare consultations. *J R Soc Med*. (2009) 102:235–43. 10.1258/jrsm.2009.090013 19531618 PMC2697041

[24] Parikh P Jaisinghani P Kaloth S Konakanchi A Yanamala N Kim S. Enhancing patient understanding of hospitalization and post-discharge needs: the impact of physician-led verbal communication and teach-back method. *J Gen Intern Med*. (2025) 40:2103–10. 10.1007/s11606-025-09510-w 40268834 PMC12325820

[25] Valdes LM Sobrin L. Uveitis therapy: the corticosteroid options. *Drugs*. (2020) 80:765–73. 10.1007/s40265-020-01314-y 32350761

[26] Suhler EB Thorne JE Mittal M Betts KA Tari S Camez A Corticosteroid-related adverse events systematically increase with corticosteroid dose in noninfectious intermediate, posterior, or panuveitis: post Hoc Analyses from the VISUAL-1 and VISUAL-2 Trials. *Ophthalmology*. (2017) 124:1799–807. 10.1016/j.ophtha.2017.06.017 28689898

[27] Besagar S Yonekawa Y Sridhar J Finn A Padovani-Claudio DA Sternberg P Association of socioeconomic, demographic, and health care access disparities with severe visual impairment in the US. *JAMA Ophthalmol*. (2022) 140:1219–26. 10.1001/jamaophthalmol.2022.4566 36326732 PMC9634598

[28] Lai Y Wu Y Chai C Yen CC Ho Y Eng TC The effect of patient education and telemedicine reminders on adherence to eye drops for glaucoma. *Ophthalmol Glaucoma*. (2020) 3:369–76. 10.1016/j.ogla.2020.05.005 32980041

[29] Kharod BV Johnson PB Nesti HA Rhee DJ. Effect of written instructions on accuracy of self-reporting medication regimen in glaucoma patients. *J Glaucoma*. (2006) 15:244–7. 10.1097/01.ijg.0000212213.18018.8f 16778648

[30] Thevarajah R Haputhanthrige IU Misbahunnisa MY Galappaththy P. Patients’ knowledge about medicines improves when provided with written compared to verbal information in their native language. *PLoS One*. (2022) 17:e0274901. 10.1371/journal.pone.0274901 36315507 PMC9621412

