# Supplementary material for: Medication adherence rates in patients with ocular inflammatory disease
Source: Front Med (Lausanne). 2026 Feb 17;13:1745392. doi: 10.3389/fmed.2026.1745392 (PMC12953074; doi:10.3389/fmed.2026.1745392)
Supplement: Supplementary file 1 [file Table_1.docx]

**Supplementary Table 1: Logistic regression results for the effect of patient demographics on adherence**

| **Variable** | **Category** | **Good Adherence (n=14)** | **Poor Adherence (n=61)** | **Univariate model** | | **Multivariate Model** | |
| --- | --- | --- | --- | --- | --- | --- | --- |
|  |  |  |  | **OR (95% CI)** | **p value** | **OR (95% CI)** | **p value** |
| Age | >70 | 2 (14.3%) | 20 (32.8%) | 2.927 (0.597-14.346) | 0.210 |  |  |
|  | <70 (Ref) | 12 (85.7%) | 41 (67.2%) | 1 (Ref) | - |  |  |
| Gender | Female | 7 (50.0%) | 34 (55.7%) | 1.259 (0.394-4.029) | 0.771 |  |  |
|  | Male (Ref) | 7 (50.0%) | 27 (44.2%) | 1 (Ref) | - |  |  |
| Language | Mandarin | 3 (21.4%) | 30 (49.1%) | 3.548 (0.900-13.988) | 0.070 |  |  |
|  | English (Ref) | 11 (78.6%) | 31 (50.8%) | 1 (Ref) | - |  |  |
| Ethnicity | Malay | 2 (14.3%) | 4 (6.6%) | 0.377 (0.061-2.345) | 0.936 |  |  |
|  | Indian | 2 (14.3%) | 3 (4.9%) | 0.283 (0.042-1.916) | 0.219 |  |  |
|  | Others | 0 (0.0%) | 1 (1.6%) | NA | NA |  |  |
|  | Chinese (Ref) | 10 (71.4%) | 53 (86.9%) | 1 (Ref) | - |  |  |
| Education | Up to University | 8 (50.0%) | 50 (82.0%) | 3.409 (0.983-11.823) | 0.053 | 2.019 (0.485-8.409) | 0.334 |
|  | University (Ref) | 6 (50.0%) | 11 (18.0%) | 1 (Ref) | - |  |  |
| Income* | <3k | 5 (45.5%) | 32 (61.5%) | 1.920 (0.517-7.128) | 0.339 |  |  |
|  | >=3k (Ref) | 6 (54.4%) | 20 (38.4%) | 1 (Ref) | - |  |  |
| Household | Alone | 3 (21.4%) | 7 (11.4%) | 0.475 (0.106-2.130) | 0.384 |  |  |
|  | Not Alone (Ref) | 11 (78.6%) | 54 (88.5%) | 1 (Ref) | - |  |  |
| Insurance | Private or Corporate | 9 (64.3%) | 40 (65.6%) | 1.481 (0.138-15.941) | 0.746 |  |  |
|  | Government | 4 (28.6%) | 18 (29.5%) | 1.500 (0.122-118.441) | 0.751 |  |  |
|  | None/Unsure (Ref) | 1 (7.1%) | 3 (4.9%) | 1 (Ref) | - |  |  |

*Patients who did not disclose income or highest education level were excluded from the analysis
