# Supplementary material for: Medication adherence rates in patients with ocular inflammatory disease
Source: Front Med (Lausanne). 2026 Feb 17;13:1745392. doi: 10.3389/fmed.2026.1745392 (PMC12953074; doi:10.3389/fmed.2026.1745392)
Supplement: Supplementary file 2 [file Table_2.docx]

**Supplementary Table 2: Univariate logistic regression results for the effect of medication and appointment frequency on adherence**

| **Variable** | **Category** | **Good Adherence (n=14)** | **Poor Adherence (n=61)** | **OR (95% CI)** | **p value** |  |
| --- | --- | --- | --- | --- | --- | --- |
| Number of medications | 1.00 | 4 (28.6%) | 14 (23.0%) | - | 0.625 (^1^global) |  |
|  | 2.00 | 3 (21.4%) | 21 (34.4%) |  |  |  |
|  | 3.00 | 3 (21.4%) | 14 (23.0%) |  |  |  |
|  | 4.00 | 3 (21.4%) | 4 (6.6%) |  |  |  |
|  | 5.00 | 1 (7.1%) | 4 (6.6%) |  |  |  |
|  | 6.00 | 0 (0.0%) | 2 (3.3%) |  |  |  |
|  | 8.00 | 0 (0.0%) | 2 (3.3%) |  |  |  |
| Mode/s of administration | Topical only | 7 (50.0%) | 30 (49.2%) | - | 0.540 (global) |  |
|  | Oral Only | 2 (14.3%) | 8 (13.1%) |  |  |  |
|  | Subcutaneous only | 0 (0.0%) | 3 (4.9%) |  |  |  |
|  | Intravenous only | 0 (0.0%) | 1 (1.6%) |  |  |  |
|  | Topical + Oral | 4 (28.6%) | 16 (26.2%) |  |  |  |
|  | Topical + Subcutaneous | 0 (0.0%) | 2 (3.3%) |  |  |  |
|  | Oral + Subcutaneous | 1 (7.1%) | 0 (0.0%) |  |  |  |
|  | Oral + Intravenous | 0 (0.0%) | 1 (1.6%) |  |  |  |
| Type of medication | Steroids only | 5 (35.7%) | 24 (39.3%) | - | 0.350 (global) |  |
|  | Steroids + others* | 9 (64.3%) | 27 (44.3%) |  |  |  |
|  | Steroid-sparing agent only | 0 (0.0%) | 1 (1.6%) |  |  |  |
|  | Others only | 0 (0.0%) | 9 (14.8%) |  |  |  |
| Follow-up appointment frequency | >= 6 Months to a year | 3 (21.4%) | 18 (30.0%) | 1.571 (0.391-6.315) | 0.524 |  |
|  | < 6 Months (Ref) | 11 (78.6%) | 42 (70.0%) |  |  |  |

* Includes non-steroidal immunosuppressive agents, intra-ocular pressure lowering agents, anti-viral, anti-biotic or anti-fungal medication
